# Judgments of Learning Following Retrieval Practice Produce Minimal Reactivity Effect on Learning of Education-Related Materials

**DOI:** 10.3390/jintelligence11100190

**Published:** 2023-09-29

**Authors:** Wenbo Zhao, Muzi Xu, Chenyuqi Xu, Baike Li, Xiao Hu, Chunliang Yang, Liang Luo

**Affiliations:** 1 School of Social Development and Public Policy, Beijing Normal University, Beijing 100875, China; zhaowb@mail.bnu.edu.cn; 2 Institute of Developmental Psychology, Faculty of Psychology, Beijing Normal University, Beijing 100875, China; maziexu114@gmail.com (M.X.);; 3 Beijing Key Laboratory of Applied Experimental Psychology, National Demonstration Center for Experimental Psychology Education, Beijing Normal University, Beijing 100875, China; 4 State Key Laboratory of Cognitive Neuroscience and Learning, Beijing Normal University, Beijing 100875, China

**Keywords:** test-enhanced learning, JOLs, reactivity effect, learning of texts, Bayesian evidence

## Abstract

Testing (i.e., retrieval practice) is one of the most powerful strategies to boost learning. A recent study observed an incidental finding that making judgments of learning (JOLs) following retrieval practice further enhanced learning of education-related texts to a medium extent (Cohen’s *d* = 0.44) by comparison with retrieval practice itself, suggesting that making JOLs may serve as an easy-to-implement educational intervention to improve the benefits of testing. Three experiments (one pre-registered) were conducted to test the replicability of Ariel et al.’s incidental finding and to further determine whether making JOLs following retrieval practice reactively enhances the benefits of testing for text learning. The three experiments consistently provided Bayesian evidence supporting no reactivity effect of JOLs following retrieval practice, regardless of whether the replication experiments were conducted in a laboratory (Experiment 1) or online (Experiments 2 and 3), whether the stimuli were presented in the same language (Experiments 2 and 3) or not (Experiment 1), and whether participants were recruited from the sample pool (Experiment 2) or not (Experiments 1 and 3) as in the original study. These null findings imply that making JOLs cannot be utilized as a practical strategy to enhance the benefits of testing for learning of educationally related materials. Possible explanations for the null reactivity effect of JOLs following retrieval practice are discussed.

## 1. Introduction

Test-enhanced learning or the testing effect refers to the fact that testing (i.e., retrieval practice) on studied information produces superior learning gains by comparison with restudying or other learning strategies (such as note making and concept mapping) (Roediger and Karpicke 2006b; Yang et al. 2021). Besides testing, recent studies demonstrated that making judgments of learning (JOLs; i.e., prospective metamemory judgments about the likelihood of remembering studied information on a later test) can also enhance learning of some types of materials, such as related word pairs (Soderstrom et al. 2015; Witherby and Tauber 2017), word lists (Zhao et al. 2022), and visual images (Shi et al. 2023), a phenomenon known as the reactivity effect of JOLs. The current study aims to explore if making JOLs and practice testing can be combined to produce additional beneficial effects on learning of education-related materials (i.e., text passages). Below, we first review evidence of test-enhanced learning, then summarize previous findings of the reactivity effect, and finally outline the rationale of the current study.

### 1.1. Test-Enhanced Learning

Test-enhanced learning has been well-established by hundreds of studies across different research settings (e.g., laboratory and classroom), populations (e.g., children, adolescents, young and older adults), and study materials (e.g., foreign-translation pairs, text passages, lecture videos) (for reviews, see Carpenter et al. 2022; Pan and Rickard 2018; Roediger and Karpicke 2006a; Shanks et al. 2023; Yang et al. 2021). Testing can facilitate learning in different ways. For instance, testing not only consolidates long-term memory of studied information, which is known as the backward testing effect (for reviews, see Adesope et al. 2017; Rowland 2014) but also facilitates subsequent learning of new information, which is labeled as the forward testing effect (for reviews, see Pastötter and Bäuml 2014). More importantly, testing can promote knowledge transfer in service of solving applied problems in new contexts, termed test-enhanced knowledge transfer (for a review, see Carpenter 2012). These findings jointly suggest that testing is a powerful and effective strategy that should be applied across a wide range of educational contexts. Educators and learners would benefit from incorporating practice testing into their study routines to improve retention, comprehension, and knowledge transfer.

Although hundreds of studies have established the merits of testing in enhancing learning, most of them focused on the “pure” benefits of testing, with the question of how to combine other strategies or practices with testing to produce joint (and larger) learning benefits being underexplored, as claimed by a recent meta-analytic review (Yang et al. 2021). The question regarding how to combine other practices with testing to produce additional learning benefits is clearly a matter of considerable educational importance.

Several studies did find that some strategies can be utilized to boost the enhancing effect of testing (e.g., Miyatsu and McDaniel 2019). For instance, the magnitude of test-enhanced learning is larger when tests are spaced out over time than when they are implemented during a massed session, suggesting that spacing can interact with testing to jointly aid learning (Delaney et al. 2010; Dunlosky et al. 2013). It has also been shown that interleaving different types of problems or concepts during tests is more effective for consolidating long-term memory by comparison with blocking one type of problem or concept (Sana and Yan 2022). However, it should be noted that not all strategies can be combined with testing to produce additional benefits. For instance, although feature highlighting alone can facilitate natural category learning, a recent study by Kang et al. (2023) showed that provision of feature highlighting does not enhance the benefits of testing for inductive learning and even may reduce the benefits of testing in some situations.

Besides the aforementioned practices, a recent study by Ariel et al. (2021) incidentally observed that making JOLs following practice retrieval enhances the benefits of testing for text learning to a medium extent (Cohen’s *d* = 0.44), suggesting a reactivity effect of JOLs following retrieval practice.

### 1.2. Reactivity Effect

An emerging body of studies demonstrated that making JOLs can reactively change memory performance (for a review, see Double et al. 2018). For instance, Zhao et al. (2022) observed that making JOLs while studying each word substantially enhanced recognition performance for elementary school children. Witherby and Tauber (2017) demonstrated that making JOLs while studying each related word pair (e.g., *pond–frog*) enhanced cued recall performance on a test administered either 5 min or 48 h later. Similarly, Shi et al. (2023) found that making JOLs facilitated memory of visual images. These positive reactivity findings together point to a tempting inference: Making JOLs may act as a simple educational intervention to aid learning of educationally related materials, as claimed by many studies (e.g., Soderstrom et al. 2015).

Ariel et al. (2021) conducted the first study to explore whether making JOLs reactively facilitates comprehension of educationally relevant texts. In their Experiments 1, 2a, 2b, and 3, Ariel et al. asked two (JOL vs. no-JOL) groups of participants to study a scientific text, divided into five sections. After reading each section, the JOL group made either a global JOL (i.e., rating how confident they were that they understood the section they just read; Experiment 1) or several term-specific JOLs (i.e., rating how confident they were that they understood each of the concepts described in the section they just read; Experiments 2a–3). By contrast, the no-JOL group did not make JOLs after reading each section. After studying all five sections, both the JOL and no-JOL groups were tested by 12 short-answer questions (e.g., *How are minerals made?*). Inconsistent with the positive reactivity findings discussed above, Ariel et al.’s Experiments 1–3 consistently showed no significant difference in test performance between the JOL and no-JOL groups, regardless of JOL type (global vs. term-specific). These findings suggest that making JOLs produces minimal reactive influence on learning of educationally related texts.

Ariel et al. (2021) speculated that the null reactivity findings observed in their Experiments 1–3 derived from the fact that participants in the JOL group might use covert retrieval to search for appropriate cues (e.g., memory retrievability of text content) to inform JOL formation but prematurely terminate the covert retrieval process during monitoring (Son and Metcalfe 2005), in turn leading to a minimal enhancing effect of making JOLs on text comprehension (see below for detailed discussion). Additionally, Ariel et al. (2021) proposed that by comparison with overt retrieval (i.e., testing), covert retrieval elicited by the requirement of making JOLs is less effective for enhancing learning of complex materials, such as key-term definitions (Tauber et al. 2018).

Ariel et al. (2021) conducted Experiment 4 to investigate whether covert (i.e., making JOLs) and overt (i.e., practice testing) retrieval produce differential effects on text learning. In this experiment, four (JOL vs. no-JOL vs. test vs. test + JOL) groups of participants studied the same text as in Experiments 1–3. The JOL group made two or three term-specific JOLs following the reading section, whereas the no-JOL group did not make JOLs. The test group took a practice test, composed of two or three short-answer questions followed by immediate corrective feedback (i.e., correct answer), after reading each section. The test + JOL group also took a practice test with corrective feedback after reading each section. Critically, different from the test group, the test + JOL group also needed to make a term-specific JOL after they encoded each question’s corrective feedback. The final test results replicated the null reactivity finding by showing no difference in test performance between the JOL and no-JOL groups. Additionally, the test group significantly outperformed the no-JOL group, reflecting a classic testing effect. These findings are consistent with Ariel et al.’s a priori prediction: Overt retrieval (i.e., practice testing) produces better learning outcomes than covert retrieval induced by the requirement of making JOLs.

Critically, and intriguingly, Ariel et al. (2021) also observed that test performance was significantly better in the test + JOL group than in the test group. Furthermore, the difference in test performance was moderate (*d* = 0.44) and nonnegligible. As claimed by Ariel et al. (2021), this finding reflects that making JOLs may reactively enhance learning of educationally related texts when JOLs are elicited following retrieval practice (i.e., overt retrieval attempts). Put differently, this finding suggests that making JOLs may interact with practice testing to produce larger learning benefits and boost the testing effect to a medium extent. Such a phenomenon clearly has important implications to guide educational practices. For instance, if making JOLs and testing can jointly produce a stronger enhancing effect on learning of educationally related materials than testing itself, such an easy-to-implement intervention should be utilized to facilitate students’ knowledge mastery in the real classroom.

Indeed, it is reasonable to expect that making JOLs following retrieval practice may reactively enhance the benefits of testing. Although it has been widely assumed that learners make their JOLs based on the momentary accessibility (i.e., retrievability) of text content through covert retrieval (Morris 1990), many studies have disproved this assumption by showing that the accuracy of judgments of text learning (i.e., metacomprehension judgments) is quite poor. For instance, Yang et al. (2022) recently conducted a large-scale meta-analysis by integrating results from over 15,000 students across 115 studies to assess learners’ metacomprehension accuracy. The results dismayingly showed that learners’ metacomprehension accuracy is quite poor, only barely better than the accuracy based on a coin toss. This meta-analysis also showed that taking a practice test (i.e., overt retrieval) before making metacomprehension judgments substantially enhanced the relative accuracy of comprehension judgments. These findings consistently imply that learners may not automatically use covert retrieval to assess retrievability of text content when making JOLs, which might be the reason why making JOLs does not reactively enhance learning of text materials.

Even though learners may automatically use covert retrieval when making JOLs, they may suspend the covert retrieval process prematurely, leading to insufficient retrieval and producing a minimal enhancing effect of making JOLs on text learning (Son and Metcalfe 2005). An available method to stimulate individuals to use covert retrieval during the JOL-making process is to promote overt retrieval attempts before asking them to make JOLs. Supporting evidence comes from DeCaro and Thomas (2020) who showed that, when participants were explicitly asked to retrieve the target information before making JOLs, they spent more time making JOL assessments than when they were not prompted to overtly retrieve.

As discussed above, prompting overt retrieval attempts before eliciting JOLs may stimulate covert retrieval during the JOL-making process. Covert retrieval during the JOL-making process plus overt retrieval may produce superior learning gains by comparison with overt retrieval itself (e.g., Karpicke and Roediger 2007). This might be the reason why Ariel et al. observed that making JOLs itself produced no reactivity effect on text learning but making JOLs following retrieval practice produced better text learning than retrieval practice itself. Specifically, participants might not use covert retrieval or suspend covert retrieval prematurely during the JOL-making process, leading to no reactivity effect on text learning. However, when JOLs were made after overt retrieval attempts, the covert retrieval process might be stimulated by prior overt retrieval attempts, which in turn cooperated with overt retrieval to produce superior learning benefits by comparison with overt retrieval itself. Below, we term this explanation as the *enhanced covert retrieval hypothesis*.

### 1.3. Rationale and Overview of the Present Study

To our knowledge, Ariel et al.’s (2021) Experiment 4 is the first and the only experiment which explored the reactivity effect of JOLs following retrieval practice on learning of educationally relevant texts. Their incidental finding that testing and making JOLs jointly facilitate text learning clearly has considerable educational implications. However, it is premature to make any firm conclusions regarding whether making JOLs can enhance the benefits of testing for text learning based on the incidental finding from a single experiment, even though the observed effect size (*d* = 0.44) is moderate. It is possible that the reactivity finding of JOLs following retrieval practice observed by Ariel et al. (2021) is a fake phenomenon, deriving from sampling error. Hence, it is of critical importance to test the replicability of Ariel et al.’s reactivity finding of JOLs following retrieval practice, which is the primary aim of the present study. Successful replication of this incidental finding will not only help to increase our confidence in the reactivity effect of JOLs following retrieval practice but also can guide educational practices. Furthermore, establishing the existence of the reactivity effect of JOLs following retrieval practice is a pre-requisite to investigate its underlying mechanisms.

Although the enhanced covert retrieval hypothesis can readily account for the reactivity effect of JOLs following retrieval practice, this hypothesis is originally proposed here and has never been empirically tested so far. Hence, we also pre-planned to test this hypothesis if we could successfully replicate Ariel et al.’s original finding. However, as illustrated below, our Experiment 1, which was a laboratory experiment and employed a Chinese version of Ariel et al.’s experimental stimuli, failed to replicate their reactivity finding by showing no benefit of JOLs following retrieval practice. Hence, the next two experiments (Experiments 2 and 3) further tested the replicability of Ariel et al.’s reactivity finding of JOLs following retrieval practice (rather than testified the enhanced covert retrieval hypothesis).

We suspect that the failed replication might derive from many divergences between our Experiment 1 and Ariel et al.’s Experiment 4. For instance, our Experiment 1 was a laboratory experiment, conducted in China, and utilized a Chinese-translation version of texts and test questions as in Ariel et al.’s Experiment 4. By contrast, Ariel et al.’s Experiment 4 was performed online and recruited participants from mTurk, and all stimuli were presented in English. Hence, our Experiment 2 followed Ariel et al.’s Experiment 4 more closely to further test the replicability of their reactivity finding. Specifically, our Experiment 2 was also conducted online with all participants recruited from mTurk and used the exact same procedure and stimuli as in Ariel et al.’s Experiment 4. Again, Experiment 2 demonstrated a failed replication by showing no reactivity influence of JOLs following retrieval practice. Next, we conducted a pre-registered experiment (i.e., Experiment 3) to re-assess whether making JOLs following retrieval practice reactively enhances text learning. Experiment 3 was performed via Prolific Academic, recruited participants from the UK, and used the exact procedure and stimuli as in Ariel et al.’s Experiment 4. Again, it showed no reactive influence of JOLs following retrieval practice. Finally, a mini meta-analysis was performed to integrate results across the three experiments to increase statistical power to further determine the existence or absence of such an effect. The meta-analysis again showed minimal reactivity effect of JOLs following retrieval practice.

## 2. Experiment 1

The main goal of Experiment 1 was to test the replicability of the reactivity finding of JOLs following retrieval practice, incidentally documented by Ariel et al. (2021).

### 2.1. Methods

Participants. Based on the magnitude (Cohen’s d = 0.44) of the reactivity effect of JOLs following retrieval practice reported by Ariel et al. (2021), a power analysis was performed via G*power to determine the required sample size (Faul et al. 2007), which indicated that 65 participants in each group were required to detect a significant (one-tailed, α = 0.05) reactivity effect at 0.80 power. Finally, 137 participants (M age = 21.19, SD = 1.67; 125 females) were recruited from Beijing Normal University (BNU), with 69 randomly allocated to the JOL group and 68 to the no-JOL group. They were tested individually in a sound-proofed cubicle and received about CNY 15 for compensation.

All participants in all experiments signed a consent form to participate. Experiments 1–3 were approved by the Ethics Committee of BNU Faculty of Psychology.

### 2.2. Materials

We used the same science text and test questions as those in Ariel et al. (2021), which were extracted from OSF shared by the original researchers (available at https://osf.io/p4rf8/). The text and test questions were translated into Chinese. The translated text contained 817 Chinese characters in total and was composed of 5 sections. The lengths of Section 1, Section 2, Section 3, Section 4 and Section 5 were 103, 146, 183, 156, and 229 characters, respectively. Each section had a descriptive heading: 地质作用 (Geological processes), 无机物 (Inorganic substances), 结晶固体 (Crystalline solids), 元素 (Elements), and 化合物 (Compounds). There were 3 short-answer questions each for Section 2 and Section 3, and 2 each for Section 1, Section 4 and Section 5.

### 2.3. Procedure

Before the main experiment, participants undertook a practice task, in which they studied a brief text selected from Shiu and Chen (2013) and answered two short-answer questions. This practice task was administered to get participants familiar with the task procedure.

The main experimental procedure in the JOL and no-JOL groups was exactly same as those for the test + JOL and test groups in Ariel et al.’s (2021) Experiment 4. Specifically, participants in both the JOL and no-JOL groups read the text, section by section, in a self-paced procedure. Although the study procedure was self-paced, participants had to spend at least 30 s reading each section before they could move forward. After participants finished reading each section, they were instructed to answer two or three practice questions corresponding to that section (e.g., *How are minerals made?*). The test questions were presented in a fixed order as the information appeared in the text. Participants had unlimited time to answer each question, but with a constraint that they had to spend at least 10 s answering each question before moving forward. After they typed their answer into a blank box and clicked a button to submit the answer, corrective feedback (i.e., correct answer) for the question was presented on screen. Encoding of corrective feedback was self-paced, but with a constraint that participants had to study the correct answer for at least 5 s. After 5 s, a button labeled “DONE” was presented, and participants could click the button to move to the next question.

For the JOL group, immediately after the presentation of corrective feedback, participants were asked to make a term-specific JOL (e.g., “*How confident are you that you understand how minerals are made?*”). JOLs were made on a slider ranging from 0 (*not very confident*) to 100 (*very confident*). There was no time limit for making JOLs. After making a JOL, the next question was shown immediately. This cycle repeated until they answered all questions in that section. By contrast, participants in the no-JOL group only needed to answer the practice questions and encode the corrective feedback, without the requirement of making JOLs.

After participants finished the practice test on the first section, they started to study the second section. This cycle repeated until they studied all five sections. Next, participants in both groups completed a final test, composed of the same 12 short-answer questions as those used in the practice tests (e.g., “*How are minerals made?*”). In the final test, participants had to spend at least 10 s to answer each question before they could move forward. No feedback was provided in the final test. At the end of the final test, participants in both groups were asked to report how familiar they were with the text content prior to reading the text. Familiarity ratings were made on a scale ranging from 0 (*not very familiar*) to 100 (*very familiar*).

Overall, the only difference between the JOL and no-JOL groups was that participants in the JOL group needed to make a JOL following encoding each question’s corrective feedback, whereas those in the no-JOL group did not need to make JOLs.

### 2.4. Results and Discussion

Below, we report both frequentist and Bayesian statistics. All Bayesian analyses were performed via JASP (Version: 0.16.2), with all parameters set as default.

Participants’ answers in the final test were independently scored by two coders who were blind to the aim of the current study. Test scores provided by the two coders were consistent (*kappa* = 0.76; *r* = 0.88, *p* < .001, *BF*_10_ > 1000). Any divergences between the two coders were settled through group discussion. Because practice test performance was not the main research interest, participants’ answers in the practice tests were only scored by a single coder.

A two-tailed Bayesian independent *t*-test was performed on test performance in the practice tests. The results showed that there was no statistically detectable difference in practice test performance between the JOL (*M* = 74.03, *SD* = 13.75) and no-JOL (*M* = 72.92, *SD* = 13.76) groups, difference = 1.12 [−3.35, 5.78], *t*(135) = 0.48, *p* = .64, Cohen’s *d* = 0.08 [−0.25, 0.42], *BF*_10_ = 0.20.

**Figure 1:**
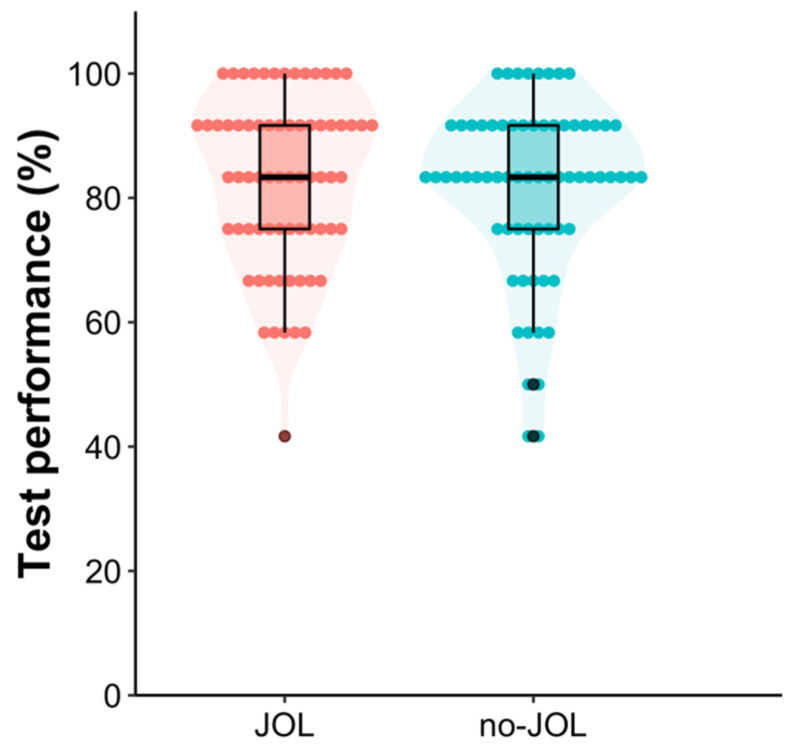
Violin and box plot of final test performance as a function of group in Experiment 1. Each dot represents a given participant’s test score.

A pre-planned one-tailed Bayesian independent *t*-test was performed on final test performance,^1^ which showed that there was no statistically detectable difference in final test performance between the JOL (*M* = 82.85, *SD* = 13.70) and no-JOL (*M* = 81.50, *SD* = 14.10) groups, difference = 1.36 [−3.34, 6.05]^2^, *t*(135) = 0.57, *p* = .29, Cohen’s *d* = 0.10 [−0.24, 0.43], *BF*_10_ = 0.30 (see Figure 1), suggesting that making JOLs following retrieval practice does not reactively facilitate text learning. This finding is inconsistent with that observed in Ariel et al.’s (2021) Experiment 4.

Another two-tailed Bayesian independent *t*-test showed that there was no detectable difference in familiarity ratings between the JOL (*M* = 36.09, *SD* = 26.08) and no-JOL (*M* = 34.28, *SD* = 30.87) groups, difference = 1.81 [−7.84, 11.46], *t*(135) = 0.37, *p* = .71, Cohen’s *d* = 0.06 [−0.27, 0.40], *BF*_10_ = 0.20. Across all participants, there was a significant correlation between final test performance and familiarity ratings, *r* = 0.20, *p* = .02, *BF*_10_ = 1.82, indicating that the greater the familiarity, the better the final performance.

In the JOL group, the average JOL was 69.37 (*SD* = 16.89), which was significantly lower than final test performance, difference = −13.48 [−17.87, −9.08], *t*(68) = −6.12, *p* < .001, Cohen’s *d* = −0.74 [−1.00, −0.47], *BF*_10_ > 1000, indicating that participants were overall underconfident about their memory performance. For each participant in the JOL group, we calculated a gamma (*G*) correlation to quantify the relative accuracy of JOLs. Thirteen participants’ data were excluded from *G* correlation analysis because of constant JOLs or test scores, making it impossible to calculate the *G* value. Among the remaining 56 participants, the relative accuracy of their JOLs (*M* of *G* = 0.39, *SD* = 0.54) was significantly greater than the chance level (0), difference = 0.39 [0.25, 0.54], *t*(55) = 5.42, *p* < .001, Cohen’s *d* = 0.72 [0.43, 1.02], *BF*_10_ > 1000, indicating that participants could metacognitively discriminate well-mastered concepts from poorly mastered ones.

## 3. Experiment 2

Experiment 1 used Chinese participants and stimuli and failed to replicate the reactivity finding documented by Ariel et al.’s (2021) Experiment 4. A possible reason for the failed replication is that our Experiment 1 used a different participant sample (i.e., Chinese participants) and the stimuli were presented in a different language. Furthermore, our Experiment 1 was a laboratory experiment, whereas Ariel et al.’s (2021) Experiment 4 was conducted online. In Experiment 2, we used the same participant sample (i.e., mTurk workers) and the same language (i.e., English) as those in Ariel et al.’s (2021) Experiment 4 to further test the replicability of Ariel et al.’s reactivity finding of JOLs following retrieval practice.

### 3.1. Methods

Participants. As in Experiment 1, the sample size was set to 65 participants in each group. Following Ariel et al. (2021), we recruited participants from mTurk and restricted recruitment to those who had completed 1000 or more HITs, with an acceptance rate greater than 95%, and located in the USA. Finally, 165 participants were recruited and randomly assigned to the JOL and no-JOL groups. They received USD 1.5 for compensation.

Among the 165 participants, data from 88 participants were excluded from analyses because they did not answer any questions correctly in the final test^3^ or reported that they used notes or searched question answers from the Internet during the final test, leaving final data only from 77 participants, with n = 39 in the JOL and n = 38 in the no-JOL group. To mitigate potential concerns about data exclusion, below we also provide results from all 165 participants, and the result pattern is exactly same as that of the 77 saved participants. We acknowledge that the final saved sample size (i.e., 77 participants in total) was smaller than pre-planned. To further mitigate worry about statistical power issues, a meta-analysis was performed after Experiment 3 (see below for details).

### 3.2. Materials and Procedure

The materials and procedure were the same as those in Ariel et al.’s Experiment 4. At the end of the final test, participants reported their familiarity with the text and whether they used notes or the Internet to answer test questions during the final test.

### 3.3. Results and Discussion

Participants’ answers in the final test were independently scored by two coders who were blind to the aim of the current study. Test scores provided by the two coders were consistent (*kappa* = 0.79; *r* = 0.92, *p* < .001, *BF*_10_ > 1000). Any divergences between the two coders were settled through group discussion. Practice test performance was scored by a single coder.

**Figure 2:**
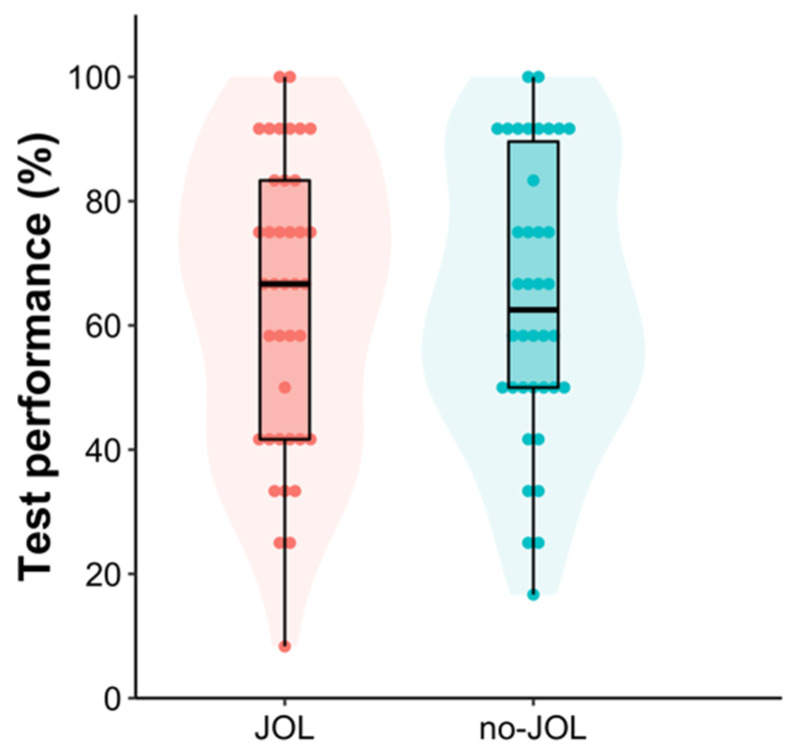
Violin and box plot of final test performance as a function of group in Experiment 2. Each dot represents a given participant’s test score.

In the practice tests, there was no detectable difference in test performance between the JOL (*M* = 52.56, *SD* = 23.35) and no-JOL (*M* = 56.58, *SD* = 23.98) groups, difference = −4.02 [−14.76, 6.73], *t*(75) = −0.74, *p* = .46, Cohen’s *d* = −0.17 [−0.62, 0.28], *BF*_10_ = 0.30. Of critical interest, the results from a one-tailed Bayesian independent *t*-test demonstrated that the JOL group’s final test performance (*M* = 63.46, *SD* = 23.70) was not different from that of the no-JOL group (*M* = 64.25, *SD* = 22.84), difference = −0.79 [−11.36, 9.78], *t*(75) = −0.15, *p* = .56, Cohen’s *d* = −0.03 [−0.48, 0.41], *BF*_10_ = 0.21 (see Figure 2), replicating the null reactivity finding observed in Experiment 1. To mitigate potential concerns about data exclusion, we conducted another one-tailed Bayesian independent *t*-test, in which the data from all 165 participants were included. The results showed the same pattern: The JOL group’s final test performance (*M* = 37.11, *SD* = 33.73) was about the same as that in the no-JOL group (*M* = 33.43, *SD* = 32.90), difference = 3.69 [−6.56, 13.92], *t*(163) = 0.71, *p* = .24, Cohen’s *d* = 0.11 [−0.20, 0.42], *BF*_10_ = 0.32.

Intriguingly, familiarity ratings were higher in the JOL (*M* = 55.72, *SD* = 34.47) than in the no-JOL (*M* = 39.97, *SD* = 28.11) group, difference = 15.74 [1.45, 30.04], *t*(75) = 2.19, *p* = .03, Cohen’s *d* = 0.50 [0.05, 0.95], although the Bayesian evidence for this difference was anecdotal, *BF*_10_ = 1.83. We assume that the superior text familiarity in the JOL group might result from sampling error because participants were randomly allocated and the Bayesian evidence for this difference was weak. There was no significant correlation between familiarity ratings and final test performance, *r* = −0.13, *p* = .26, *BF*_10_ = 0.27.

We next conducted a Bayesian analysis of covariance (ANCOVA), with group (JOL vs. no-JOL) as the independent variable, familiarity ratings as the covariate, and final test performance as the dependent variable. This analysis was performed to determine whether making JOLs following retrieval practice reactively enhanced text learning when the difference in familiarity ratings between groups was controlled. The results showed no main effect of study method, *F*(1, 74) = 0.02, *p* = .90, *η*_p_^2^ < 0.001, *BF*_10_ = 0.24, re-confirming no reactivity effect of JOLs following retrieval practice on text learning. Furthermore, including familiarity ratings did not enhance the goodness of model fitting, *F*(1, 74) = 1.26, *p* = .27, *η*_p_^2^ = 0.02, *BF*_10_ = 0.41. Overall, regardless of whether the difference in familiarity ratings was controlled or not, the results always supported the claim that making JOLs following retrieval practice does not produce additional benefits for text learning.

In the JOL group, the average JOL was 74.75 (*SD* = 15.59), which was significantly higher than final test performance, difference = 11.28 [1.71, 20.86], *t*(38) = 2.39, *p* = .02, Cohen’s *d* = 0.38 [0.05, 0.71], *BF*_10_ = 2.11, indicating that participants were overall overconfident about their memory performance. We assume that the divergent findings of overconfidence in Experiment 2 and underconfidence in Experiment 1 came from the fact that final test performance was overall lower in Experiment 2 (conducted online) than in Experiment 1 (conducted in a laboratory). Two participants’ data were excluded from *G* correlation analysis because they provided constant JOLs for all items. Among the remaining 37 participants, the relative accuracy of their JOLs (*M* of *G* = 0.28, *SD* = 0.49) was significantly greater than the chance level (0), difference = 0.28 [0.12, 0.45], *t*(36) = 3.54, *p* < .001, Cohen’s *d* = 0.58 [0.23, 0.93], *BF*_10_ = 28.20.

## 4. Experiment 3

Experiment 2 followed Ariel et al.’s Experiment 4 more closely (such as recruiting mTurk workers as participants and presenting all stimuli in English) but still failed to replicate Ariel et al.’s reactivity finding of JOLs following retrieval practice. By comparing results between Experiments 1 and 2, we noticed that final test performance was substantially lower in Experiment 2 than in Experiment 1, which might result from low task motivation of mTurk participants. In addition, many participants reported that they completed the final test with help from notes or the Internet. Such poor data quality might have contributed to Experiment 2’s failed replication, even though this possibility should be small because the results always support the null hypothesis regardless of excluding some participants’ data from analyses or not. We next conducted a pre-registered online experiment (i.e., Experiment 3) to address the poor data quality issue and to further test the replicability of Ariel et al.’s reactivity finding of JOLs following retrieval practice. Pre-registration information is available at https://osf.io/zj2ys.

To address the poor data quality issue, Experiment 3 recruited participants from another online platform: Prolific Academic. Different from mTurk, Prolific Academic has a high-quality participant population (Palan and Schitter 2018), and Prolific Academic respondents provide higher-quality responses (Peer et al. 2017). In addition, before initiating the main task, Experiment 3 explicitly warned participants not to use notes or the Internet to aid test performance and that their test performance would not affect task compensation.

### 4.1. Methods

Participants. Following Experiments 1 and 2, the sample size for Experiment 3 was set at 65 participants per group. Finally, we gathered data from 132 Prolific Academic participants (M age = 33.06, SD = 7.97; 59.9% female), with n = 66 randomly each allocated to the JOL and no-JOL groups. No participants met the pre-registered exclusion criteria, and hence all participants’ data were included in analyses. They received GBP 1.70 as compensation.

### 4.2. Materials and Procedure

The materials and procedure were the same as those in Ariel et al.’s Experiment 4. At the end of the final test, participants reported their familiarity with the text and whether they used notes or the Internet to answer test questions during the final test.

### 4.3. Results and Discussion

Note that all reported analyses were performed as pre-registered. Participants’ answers in the final test were independently scored by two coders who were blind to the aim of the current study. Test scores provided by the two coders were consistent (*kappa* = 0.73; *r* = 0.82, *p* < .001, *BF*_10_ > 1000). Any divergences between the two coders were settled through group discussion. Practice test performance was scored by a single coder.

**Figure 3:**
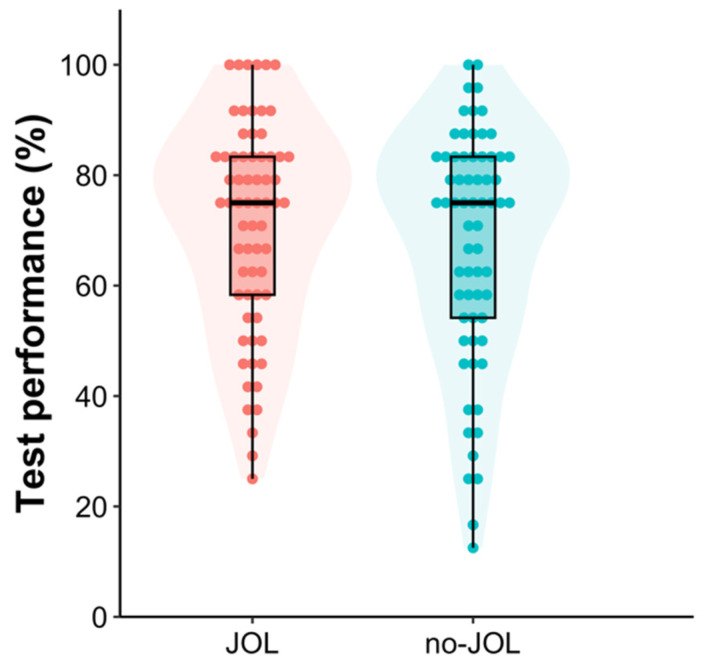
Violin box plots of test performance as a function of group in Experiment 3. Each dot represents a given participant’s test score.

In the practice tests, there was no detectable difference between the JOL (*M* = 57.64, *SD* = 18.56) and no-JOL (*M* = 55.56, *SD* = 17.74) groups, difference = 2.08 [−4.17, 8.34], *t*(130) = 0.67, *p* = .51, Cohen’s *d* = 0.12 [−0.23, 0.46], *BF*_10_ = 0.23. The results from a one-tailed Bayesian independent *t*-test demonstrated that there was no detectable difference in final test performance between the JOL (*M* = 71.02, *SD* = 19.16) and no-JOL (*M* = 67.61, *SD* = 21.19) groups, difference = 3.41 [−3.55, 10.37], *t*(130) = 0.97, *p* = .17, Cohen’s *d* = 0.17 [−0.17, 0.51], *BF*_10_ = 0.47 (see Figure 3), replicating the results of Experiments 1 and 2.

Out of expectation, text familiarity ratings were higher in the JOL (*M* = 39.49, *SD* = 29.29) than in the no-JOL (*M* = 24.55, *SD* = 25.74) group, difference = 14.94 [5.45, 24.43], *t*(130) = 3.11, *p* = .002, Cohen’s *d* = 0.54 [0.19, 0.89], *BF*_10_ = 13.90. Familiarity ratings positively correlated with final test performance, *r* = 0.38, *p* < .001, *BF*_10_ > 1000.

To address the difference in text familiarity between the two groups, a Bayesian ANCOVA was performed, with group (JOL vs. no-JOL) as the independent variable, familiarity ratings as the covariate, and final test performance as the dependent variable. The results showed no main effect of study method, *F*(1, 129) = 0.04, *p* = .85, *η*_p_^2^ < 0.001, *BF*_10_ = 0.29, re-confirming no reactivity effect of JOLs following retrieval practice on text learning. Familiarity ratings significantly predicted final test performance, *F*(1, 129) = 20.55, *p* < .001, *η*_p_^2^ = 0.14, *BF*_10_ > 1000.

In the JOL group, the average JOL (*M* = 67.15, *SD* = 21.28) tended to be lower than final test performance, difference = −3.87 [−8.05, 0.31], *t*(65) = −1.85, *p* = .07, Cohen’s *d* = −0.23 [−0.47, 0.02], *BF*_10_ = 0.67, indicating that participants were relatively underconfident about their memory performance. In the JOL group, six participants’ data were excluded from *G* correlation analysis because they either provided constant JOLs for all items or correctly answered all questions in the final test. Among the remaining 60 participants, the relative accuracy of their JOLs (*M* of *G* = 0.41; *SD* = 0.48) was significantly greater than the chance level (0), difference = 0.41 [0.29, 0.53], *t*(59) = 6.60, *p* < .001, Cohen’s *d* = 0.85 [0.55, 1.15], *BF*_10_ > 1000.

## 5. Meta-Analysis

Experiments 1–3 consistently showed Bayesian evidence supporting the null hypothesis that making JOLs following retrieval practice does not further enhance the magnitude of test-enhanced learning. However, it should be acknowledged that the sample sizes in all three experiments were determined according to the original effect size reported by Ariel et al.’s (2021) Experiment 4. It is frequently argued that primary effect sizes are often inflated. Hence, deciding the required sample size according to the primary effect size reported by Ariel et al. (2021) might render our sample sizes underpowered to detect a “true reactivity effect” of making JOLs following retrieval practice. Put differently, the null findings in our Experiments 1–3 might be falsely negative due to underpowered sample sizes, even though all three experiments showed clear Bayesian evidence supporting the null hypothesis.

To mitigate worry about statistical power issues, we conducted a random-effects meta-analysis, which integrated results across all three experiments (i.e., data across 346 participants) to further determine whether making JOLs following retrieval practice enhances the magnitude of test-enhanced learning. Specifically, we first translated all Cohen’s *d*s (i.e., standardized mean differences in final test performance between the JOL and no-JOL conditions) into Hedges’ *g*s and then performed a random-effect meta-analysis via JASP.

**Figure 4:**
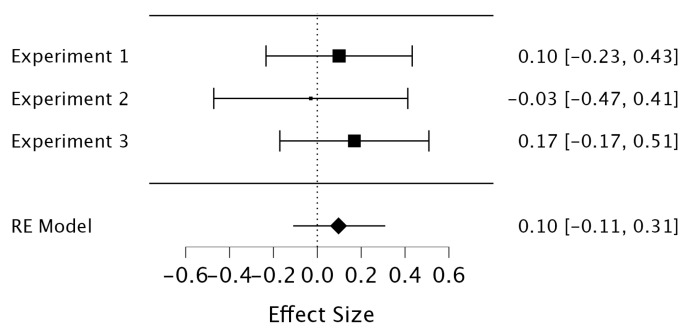
Forest plot depicting the meta-analytic results.

As shown in Figure 4, the results again showed no detectable difference in final test performance between the JOL and no-JOL conditions, Hedges’ *g* = 0.10 [−0.11, 0.31], *p* = .36. To determine the robustness of the meta-analytic finding, we also performed a Bayesian random-effects meta-analysis via JASP, with all parameters set as default. The results again showed clear evidence supporting no reactivity effect of making JOLs following retrieval practice, *BF*_10_ = 0.19.

## 6. General Discussion

Although testing has been repeatedly established as an effective learning strategy, how to combine other strategies or practices with testing to produce additional learning benefits has been underexplored (Yang et al. 2021). Ariel et al. (2021) recently documented that making JOLs following retrieval practice enhanced the benefits of testing to a medium extent. Considering the important educational implications of this incidental finding, the current study was carried out to test its replicability.

Experiment 1 recruited Chinese participants, used Chinese versions of the stimuli, and adopted the same experimental procedure as in Ariel et al.’s Experiment 4. Different from Ariel et al.’s Experiment 4, our Experiment 1 showed clear Bayesian evidence supporting no reactivity effect of JOLs following retrieval practice on learning of educationally related texts. We suspected that this failed replication might result from divergences (e.g., differences in research setting, language, and participant sample) between our Experiment 1 and Ariel et al.’s Experiment 4. Hence, we conducted another experiment (Experiment 2) to further test the replicability of the reactivity finding observed in Ariel et al.’s Experiment 4. Experiment 2 followed Ariel et al.’s Experiment 4 more closely by recruiting participants from the same participant pool (i.e., mTurk) and using the exact same stimuli. However, again, Experiment 2 showed Bayesian evidence supporting no reactivity effect of JOLs following retrieval practice. It should be acknowledged that Experiment 2’s data quality was poor, which might contribute to the failed replication. To enhance data quality, Experiment 3 (pre-registered) recruited participants from another online platform (i.e., Prolific Academic). Consistent with Experiments 1 and 2, Experiment 3 again demonstrated clear Bayesian evidence supporting the absence of the reactivity effect of JOLs following retrieval practice. Furthermore, the meta-analysis, which integrated results across Experiments 1–3 to increase statistical power, again showed clear Bayesian evidence supporting no reactivity effect of JOLs following retrieval practice.

The consistent Bayesian findings from Experiments 1–3 and meta-analysis jointly point to the strong conclusions (1) that making JOLs following retrieval practice does not reactively enhance the benefits of testing for learning of education-related texts and (2) that the reactivity effect of JOLs following retrieval practice incidentally documented by Ariel et al. (2021) is a fake phenomenon, which might result from sampling error. The null reactivity effect of JOLs following retrieval practice documented here and the null reactivity effect of JOLs observed by Ariel et al.’s (2021) Experiments 1–3 together suggest that making JOLs cannot be utilized as a practical intervention to facilitate text learning.

Besides Ariel et al. (2021) and the current study, another recent study explored whether making JOLs reactively facilitates learning of another type of educationally representative material, that is, general knowledge facts (Schäfer and Undorf 2023). In the same line, Schäfer and Undorf (2023) found no reactivity effect of JOLs on learning of general knowledge facts. It is intriguing that making JOLs reactively enhances learning of simple materials, such as related word pairs (Witherby and Tauber 2017), word lists (Zhao et al. 2022), and visual images (Shi et al. 2023), but does not affect learning of complex materials such as text passages (Ariel et al. 2021) and general knowledge facts (Schäfer and Undorf 2023). The current study further established that making JOLs following overt retrieval attempts, which is expected to prompt covert retrieval during monitoring (DeCaro and Thomas 2020), still fails to facilitate text learning.

Two possible explanations are available to account for the divergent reactivity effects on learning of simple and complex materials. The first is that covert retrieval induced by the requirement of making JOLs may be less effective for enhancing learning of complex materials than for simple materials (Ariel et al. 2021; Tauber et al. 2018). Consistent with this explanation, Smith et al. (2013) found that covert retrieval benefits retention of word lists as much as overt retrieval. Similarly, Putnam and Roediger (2013) observed that covert retrieval produced an equivalent enhancing effect on learning of related word pairs to overt retrieval. By contrast, Tauber et al. (2018) demonstrated that only overt retrieval, but not covert retrieval, enhanced learning of key-term definitions. Along the same line, Krumboltz and Weisman (1962) found that only overt retrieval, but not covert retrieval, facilitated learning of textbook contents.

Another possible explanation for the divergent reactivity effects on learning of simple and complex materials is the natural difference between simple (e.g., word pairs and word lists) and complex (e.g., text passages and general knowledge facts) materials. As noted by Schäfer and Undorf (2023), word pairs and word lists used in many previous reactivity studies typically lack interitem relations (i.e., relations among different items), whereas knowledge points stated in text passages are always interrelated and mastery of these materials involves constructing relations among pieces of information (Rohrer et al. 2020). Two recent studies consistently showed that although making JOLs reactively enhances item memory (i.e., memory of the item itself), it concurrently impairs interitem relational memory (i.e., memory of interitem relations) (Zhao et al. 2023). The negative reactivity effect on interitem relational memory (i.e., knowledge integration) might be one of the reasons why making JOLs does not aid learning of educationally related materials.

Several limitations and future research directions should be elaborated. Given that the current study did not explore whether making JOLs itself (without retrieval practice) reactively enhances text learning and it is insufficient to draw firm conclusions based on the single study by Ariel et al. (2021), future research should further determine whether the reactivity effect of making JOLs on text learning truly exists. As aforementioned, two possible explanations are available to account for the divergent reactivity effects on memory of simple and complex materials, but neither of them has been empirically tested. Future research is encouraged to do so. In addition, it is still premature to conclude no reactivity effect on learning of all types of educationally related materials based on existing findings. Future research could profitably explore if making JOLs (including making JOLs following retrieval practice) facilitates learning of other types of educationally related materials, such as key-term definitions, statistical concepts, and natural categories (Kubik et al. 2022). As discussed above, one of the possible reasons for the null reactivity effect of making JOLs on text learning might derive from the fact that making JOLs disrupts the information integration process. Previous studies provided suggestive evidence that, by comparison with JOLs, asking learners to make judgments about their mental model (i.e., judgments of inferencing; JOI) has the tendency to alert them to the gaps in their mental model of the text’s referent and thus promotes text comprehension (e.g., Nguyen and McDaniel 2016). Hence, another direction for future research is to investigate whether making JOIs (either with or without retrieval practice) can reactively facilitate text comprehension. Finally, but importantly, the study–test interval (i.e., retention interval) was quite short in the current study, and the same was true in Ariel et al. (2021). It has been well-established that retrieval-enhanced learning is most effective after long delays (e.g., Rowland 2014; Roediger and Karpicke 2006b). Future research needs to investigate whether making JOLs following retrieval practice (which is expected to elicit covert retrieval) can facilitate long-term retention of text materials.

Overall, our Experiments 1–3 consistently provided Bayesian evidence supporting the absence of reactivity influence of making JOLs following retrieval practice on learning of educationally related texts. Research findings from the current study and those from Ariel et al. (2021) and Schäfer and Undorf (2023) together suggest that the beneficial effect of making JOLs on learning of educationally related materials (e.g., text passages and general knowledge facts) tends to be limited, although making JOLs can facilitate learning of some types of simple materials.

## Data Availability

The data contained in this project are publicly available at Open Science Framework (https://osf.io/4wj7b/).

## Notes

1. Here, we implemented a one-tailed (rather than two-tailed) *t*-test because we primarily aimed to replicate the reactivity finding of JOLs following retrieval practice documented by Ariel et al.’s (2021) Experiment 4.

2. Following a reviewer’s suggestion, here we report the conventional two-tailed confidence interval, even though the *t*-test was one-tailed.

3. Recall that participants had already taken practice tests and received corrective feedback before the final test, and the practice tests and the final test were composed of the same questions. Hence, it is unlikely that a given participant could not answer any questions correctly in the final test if he or she paid attention to the task. Accordingly, the participants who did not answer any questions correctly in the final test were removed from analyses.
